# Consumer choices regarding genome-edited food crops: lessons from Japan

**DOI:** 10.3389/fgeed.2025.1672358

**Published:** 2025-09-23

**Authors:** Tetsuya Ishii

**Affiliations:** Office of Health and Safety, Hokkaido University, Sapporo City, Japan

**Keywords:** genome editing, food crop, regulation, consumer choice, marketing, labeling, organic food, risk communication

## Abstract

Japan has rapidly deregulated certain types of agricultural genome editing, yet the societal integration of these products warrants further investigation. This paper analyzed the sale and people’s perception of genome-edited food crops in Japan after reviewing the regulatory framework. Of four genome-edited crops approved as non-genetically modified organism, only one is sold online to consumers who credit safety information and perceive usefulness. Some consumers express deep safety concern, advocating mandatory labeling. The majority of people are not sufficiently aware of genome editing. To enhance informed consumer choices of genome-edited food crops, it is crucial to share visions in society, hold risk communication for mutual understanding, and maintain clear labels, including organic food standards.

## 1 Introduction

Genome editing technologies facilitated targeted genetic modifications in plant and animal cells (Gaj et al., 2013). Among them, CRISPR-Cas9, which adopts an RNA-guided nuclease, was awarded the Nobel Prize in Chemistry 2020, and the award acknowledged that CRISPR-Cas9 can be used to breed useful crop varieties (Nobel Prize Organisation, 2020). There has been a regulatory issue regarding the treatment of genome-edited organisms under the preexisting genetically modified organism (GMO) regulations (Dederer et al., 2019). After targeted mutagenesis with carefully designed Cas9 nuclease, cells without exogenously introduced genetic materials in their genome can be selected for use (Woo et al., 2015), and several countries have exempted that genome-edited organisms having no exogenous genetic materials from their GMO regulations (Atimango et al., 2024). In 2019, Japan also deregulated genome-edited organisms having no exogenous nucleic acid from the Law Concerning the Conservation and Sustainable Use of Biological Diversity through Regulations on the Use of Living Modified Organisms (LMOs; Japan’s legal term for GMOs), the so-called Cartagena Law (MOE, 2019). In 2021, a Japanese company marketed a genome-edited *Solanum lycopersicum* (tomato) variety with a higher content of gamma-aminobutyric acid (GABA), and this has been reported as a successful case (Waltz, 2022). However, some Japanese people have expressed deep concern about the sale of this genome-edited tomato in Japan (CUJ, 2021, OK Seed PJ, 2021). It is therefore necessary to examine how Japanese people view such products. This article reviews the Japan’s regulatory framework for agricultural genome editing, and analyzes the sale of, and people’s attitude to genome-edited food crops in Japan. The analyzes reveal that, Japan is currently far from full social acceptance of agricultural genome editing. Based on lessons in Japan, key tasks to improve consumer choices concerning genome-edited food are proposed.

## 2 Japan’s regulatory framework for agricultural genome editing

**FIGURE 1:**
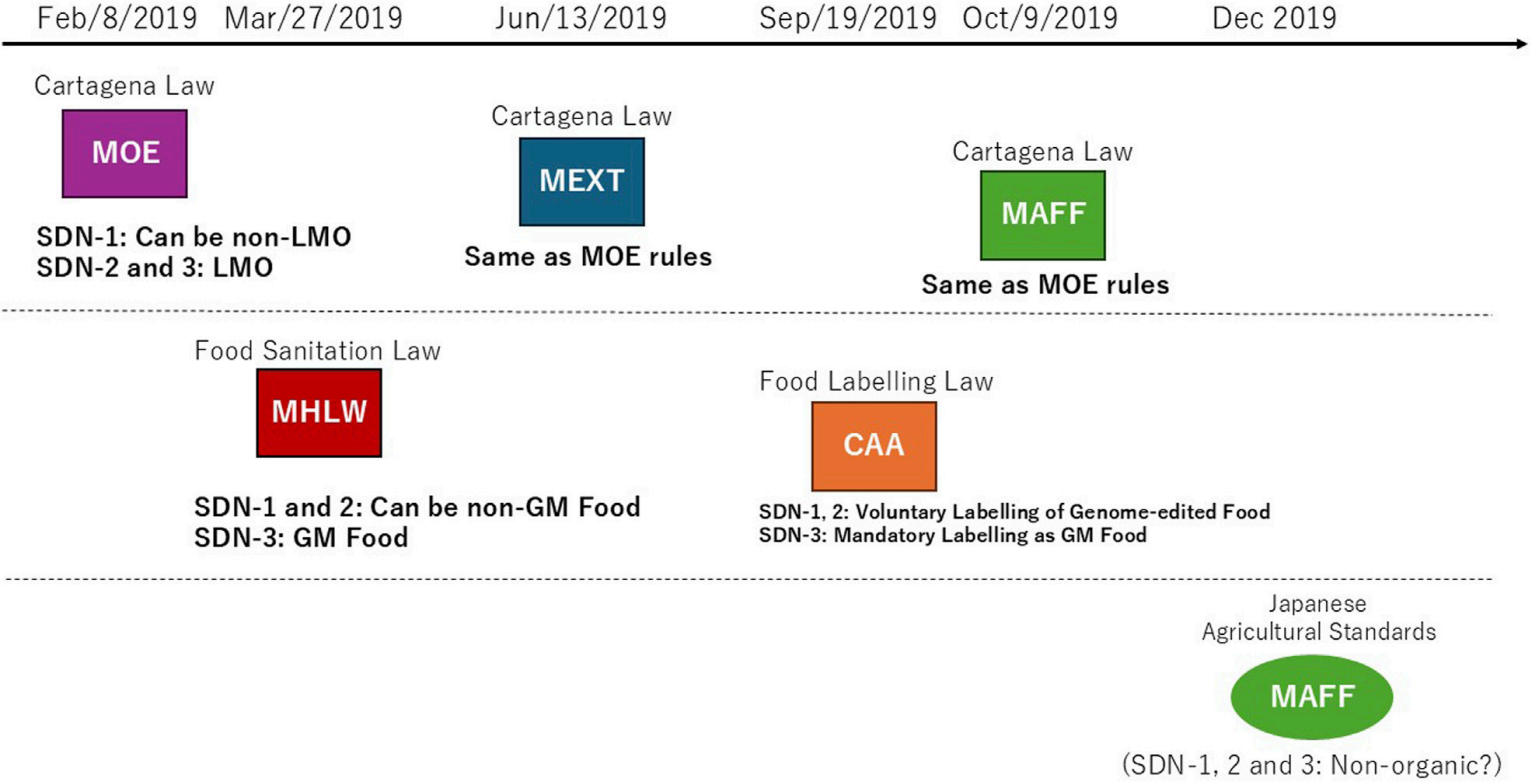
Japan’s regulatory framework for agricultural genome editing. According to the Integrated Innovation Strategy (June 2018), five competent ministries determined their rules on the treatment of agricultural genome editing, respectively. These ministerial rules fall into two groups. Rules (boxes with frame border) under Cartagena Law (Upper) exclude SDN-1 from LMO if a genome-edited plant variety has no exogenous DNA in the genome. The uses of genome editing for research and agriculture are also reviewed from a standpoint of biological diversity. Rules under Food Sanitation Law that exclude SDN-1 and SDN-2 if a genome-edited plant variety from which a food product is derived has no exogenous DNA in the genome. Genome-edited food is also reviewed from the standpoints of allergenicity and toxicity, as well as changes in metabolic systems. Cartagena Law, Law Concerning the Conservation and Sustainable Use of Biological Diversity through Regulations on the Use of Living Modified Organisms; MOE, Ministry of the Environment; MEXT, Ministry of Education; Sports; Science and Technology; MHLW, Ministry of Health; Labour; and Welfare; MAFF, Ministry of Agriculture; Fishery; and Forest; CAA, Consumer Affair Agency; SDN, Site-directed Nuclease; LMO, Living Modified Organism; GM, Genetically Modified. Notice documents (Upper): The director-general of MOE Nature Conservation Bureau No. 1902081 on 8 February 2019, the director-general of MEXT Director-General, Research Promotion Bureau No. 100 on 13 June 2019, the director-general of MAFF Director-General, Consumer Affairs and Safety Bureau No. 2743 on 9 October 2019. Rule documents (Lower): The MHLW Newly-developed Food Investigation Subcommittee, Food Hygiene Subcommittee, Pharmaceutical Affairs and Food Sanitation Council on 27 March 2019, the CAA Food Labeling Planning Division on 19 September 2019. Note that the MAFF considered excluding genome-edited food crops from organic agricultural products in Japan Agricultural Standards since December 2019; however, it is still pending.

Scholars had called for regulatory considerations of genome editing in Japan (Araki et al., 2014). In June 2018, the Cabinet Office determined the Integrated Innovation Strategy, which ordered ministries to clarify the treatments of agricultural genome editing under the Cartagena Law and resultant food products under the Food Sanitation Law that regulates GM foods produced by recombinant DNA techniques (Cabinet Office, 2018). Competent ministries had to make regulatory decisions by the end of March 2019. The process of developing the regulatory framework is reviewed, taking crop genome editing as an example (Figure 1).

The Ministry of the Environment (MOE) first considered regulatory handling of three types of genome editing: Site-directed nuclease (SDN)-1 that introduces an insertion, deletion or point mutation at a target site by DNA double-strand break and error-prone repair, SDN-2 that introduces a specific sequence replacement at a target site by introducing extracellularly prepared DNA repair template, and SDN-3 that inserts such as a full gene in a similar manner as SDN-2. In February 2019, MOE ruled that organisms resulting from SDN-1 are non-LMO, if it is proven that no exogenous nucleic acid is left in the genome, and that organisms resulting from SDN-2 or SDN-3 fall under the Cartagena Law because these involve extracellularly introducing nucleic acid (MOE, 2019). In March 2019, the Ministry of Health, Labour, and Welfare (MHLW) ruled that foods derived from organisms produced using SDN-1 or SDN-2 can be exempted from the Food Sanitation Law, and that foods derived from SDN-3 fall under the Law; explaining that food derived from SDN-1 or 2 may be indistinguishable from food produced using naturally occurring mutagenesis or conventional breeding (MHLW, 2019).

In June 2019, the Ministry of Education, Sports, Science and Technology (MEXT) issued rules regarding research and development using genome editing (MEXT, 2019), and the Ministry of Agriculture, Forestry and Fisheries (MAFF) issued their rules regarding agricultural genome editing in October 2019 (MAFF, 2019). Both MEXT and MAFF rules conform to the MOE rule that deregulates only SDN-1 from the Cartagena Law. In September 2019, the Consumer Affair Agency (CAA) issued a labeling policy of genome-edited foods under the Food Labeling Law (CAA, 2019a), which conforms to the MHLW rule that deregulates foods derived from SDN-1 or SDN-2 from the Food Sanitation Law. Namely, genome-edited foods that were approved as non-GM food are free from the mandatory labeling for GM food; however, voluntary labeling such as “genome-edited” was called for. In December 2019, MAFF considered excluding crops produced using SDN-1, 2 and 3 from the organic agricultural products by the Japanese Agricultural Standards (MAFF-JAS, 2019). Although public consultation supported the draft exclusion, the final decision has not been reached.

Thus, MOE and MHLW achieved the Integrated Innovation Strategy 2018 order by the end of fiscal year 2018, which led to regulatory rulings by MEXT, MAFF and CAA. Importantly, MOE noted that it may change the deregulation of SDN-1 if any important knowledge or findings regarding the safety of genome editing becomes available (MOE, 2019). Moreover, in addition to SDN-1 foods, MHLW deregulated SDN-2 foods, which differs from the MOE decision. At present, the treatment of genome-edited crops in the organic food standards is undecided. The implications are discussed below.

## 3 Approvals and sales of genome-edited crops in Japan

MAFF has accepted four notifications that a genome-edited crop is considered as non-LMO and has no adverse effects on biological diversity (MAFF, 2025). The notified crops were all developed using CRISPR/Cas9, demonstrating its usefulness in selective breeding (Table 1). The first notification of genome-edited tomato #87-17 by Sanatech Life Science Co., Ltd. was accepted in 2020. According to their notification, one base was inserted into *SIGAD3* (glutamate decarboxylase gene) by SDN-1, thus elevating the content of GABA that inhibits blood pressure increase. To prove that this tomato line has no exogenous DNA and no off-target mutations in the genome, Sanatech conducted two types of *in silico* analysis and one type of *in vitro* analysis. Subsequently, #87-17 was also confirmed as non-GMO in the U.S. and the Philippines (USDA, 2020; Biotech for Life, 2021). Following this, Sanatech obtained the approval of another GABA-rich tomato #206-4 in Japan. In 2023, Corteva Agriscience Japan Co., Ltd. (the Japanese subsidiary of Corteva Agriscience, Inc., headquartered in the U.S.) obtained the MAFF approval of genome-edited *Zea mays* (corn) PH1V69 having a high amylopectin content. To develop this CRISPR waxy corn, *Wx1* (waxy gene) was deleted using SDN-1 with two types of guide RNA. Corteva performed one type of *in silico* analysis and one type of *in vitro* analysis to prove the absence of exogenous DNA and off-target mutation in the corn’s genome. The genome-edited corn was also confirmed as non-GMO in the U.S. and Brazil, and as ‘non-novel’ in Canda (CBAN, 2021). Genome-edited *Solanum tuberosum* (potato) JA36 notified by J. R. Simplot Company, headquartered in the U.S., was approved as non-LMO in 2024. To increase potato’s tuber yield, SDN1 was performed, by which different deletions were introduced in all alleles of *Gn2*. Simplot performed two types of *in silico* analysis and two types of *in vitro* analysis to prove the non-existence of exogenous DNA and off-target mutations. MHLW also approved those four food crop varieties as non-GM food and safe in human consumption (MHLW, 2025) (Table 1). Among the four genome-edited crops approved in Japan, only GABA-rich tomato #87-17 has been sold (MHLW, 2025) (Table 1). The marketing of the other approved crops has not been decided. Sanatech sells the genome-edited tomato not at stores but directly to end consumers though their commerce website (Sanatech, 2024). Importantly, Sanatech labels tomato #87-17 as “improved by genome editing technology” according to the CAA rule 2019 (Sanatech, 2020). In addition, Sanatech distributed approx. 20,000 #87-17 seedlings free of charge to 5,000 people for kitchen garden cultivation (Sanatech, 2021). In response, some consumer groups have opposed the marketing and distribution of genome-edited food crops due to potential health risks and adverse effects on the environment (CUJ, 2021). Some retailers have voluntarily labeled their merchandise as “OK seed” that conveys “not genome-edited” and “No! GMO” (OK Seed PJ, 2021).

**TABLE 1:** Genome-edited food crop varieties approved as non-living modified organisms in Japan (As of July 16, 2025).

MAFF approved	Notifier	Developer	Variety	Line	Genome editing	Methods used to prove no exogenous nucleic acid and no off-target mutations in the genome	Marketing in Japan
2020/12/11	Sanatech Life Science Co., Ltd.	Same as left	Tomato highly accumulating GABA due to a partial modification in the Glutamate Decarboxylase gene (Sicilian Rouge High GABA)	#87-17	Inserting one base at the SIGAD3 gene by CRISPR-Cas9	*In silico* analysis: CRISPRdirect and Cas-OFFinder. *In vitro* analysis: Not disclosed	September 2021 (Online sale only)
2023/03/20	Corteva Agriscience Japan Co., Ltd.	Pioneer Hi Bred International Co., Ltd.	PH1V69 CRISPR-Cas9 waxy corn	ー	Deleting a region including Wx1 gene by CRISPR-Cas9	*In silico* analysis: A software linking in- house database. *In vitro* analysis: Targeted sequencing	Not marketed
2023/07/27	Sanatech Life Science Co., Ltd.	Same as left	Tomato highly accumulating GABA due to a partial modification in the Glutamate Decarboxylase gene (Esprosso High GABA)	#206-4	Same as #87-17	*In silico* analysis: CRISPRdirect and Cas-OFFinder. *In vitro* analysis: Not disclosed	Not marketed
2024/10/23	J.R. Simplot Company	Same as left	Potato with a large number of mini-tubers	JA36	Deleting 110bp at one allele and 1-2bp at three alleles of Gn2 gene by CRISPR-Cas9	In silico analysis: Cas-Designer and GuideScan. In vitro analysis: Nested PCR and targeted sequencing	Not marketed

*MAFF: ministry of agriculture, forestry and fisheries, GABA: gamma-aminobutyric acid, bp: base pairs.

*This table was created based on the information disclosed by MAFF (https://www.maff.go.jp/j/syouan/nouan/carta/tetuduki/nbt_tetuzuki.html).

*The safety in human consumption and animal feed of all the above-listed crops was confirmed by Ministry of Health, Labour and Welfare (https://www.mhlw.go.jp/stf/seisakunitsuite/bunya/kenkou_iryou/shokuhin/bio/genomed/newpage_00010.html) and MAFF (https://www.maff.go.jp/j/syouan/tikusui/siryo/ge_todokede.html).

Those ministerial approvals show that Japan’s regulatory framework has, to some extent, promoted the business development of genome-edited food corps in Japan. In addition to the single domestic company (Sanatech), two international firms also obtained the approval of their genome-edited food crops as non-LMO and non-GM food. It should also be noted that various techniques were used to demonstrate the absence of exogenous DNA and off-target mutations in the genome. Currently, only genome-edited tomato #87-17, which is labeled as genome-edited, is sold to consumers via the internet, not at food stores. Some consumer groups have deep concern about the marketing and the plant distribution of tomato #87-17, and opposition movements have developed.

## 4 Japanese people’s attitude about genome-edited crops

Table 2 summarizes the results of representative survey reports published in English regarding Japanese attitudes toward genome editing and/or genome-edited (GE) food crops. Before MOE ruled the use of genome editing in 2019, Kato-Nitta et al. performed internet surveys from 2016 to 2017 (Kato-Nitta et al., 2019). They compared lay public, molecular biology experts and experts in the other fields in risk, benefit, and value perceptions of GE crops, GM crops and crops bred by conventional techniques. Regarding GE crops, the experts in other fields were similar to molecular biology experts in benefit and value perceptions, while they were also similar to lay people in risk perception. Importantly, science literacy of lay public influenced their benefit perception but not risk or value perception of GE crops. Watanabe et al. investigated the awareness and acceptance of genome editing and related media coverage in Japan from 2016 to 2019 by collecting responses to online questionnaires (Watanabe et al., 2020). Notably, Chinese researchers announced in 2018 that they created human infants using embryo genome editing to confer resistance to HIV infection; however, the creation of genome-edited humans was regarded as an unethical reproductive experiment (Cyranoski and Ledford, 2018). Watanabe et al. concluded that this sensational announcement from China raised people’s awareness of genome editing, although such medical uses of genome editing were not associated with agricultural genome editing in Japan. Tabei et al. timely analyzed posts on the social networking service (SNS), “X” (formerly “Twitter”) by Japanese people in 2019 (Tabei et al., 2020). They observed that posts related to the ministerial rulings and relevant news had increased, and the majority of the posts regarding genome-edited food and the CAA policy that does not mandate companies to label such foods were negative. They also reported a strong demand for mandatory labeling, and noted that 90% of the posts were reposts, implying that a small number of users had acted as influencers in the negative posts. In 2020, Shinehara et al. surveyed the general public and genome editing experts and compared the two groups’ attitudes (Shineha et al., 2024). The survey results revealed that the public was taking a “wait-and-watch” attitude toward genome-edited food, and they wanted to be provided basic information about genome-edited foods and were apprehensive about risk governance systems for such foods, whereas the experts emphasized the adequacy and necessity of genome editing. Such attitudinal differences suggest a lack of risk communication regarding genome-edited food between the public and experts in Japan, which is also supported by the earlier survey reported in 2019 that people’s science literacy did not influence risk perception (Kato-Nitta et al., 2019). In 2020, Kato-Nitta et al. performed an internet survey of Japanese, German, and U.S. people (Kato-Nitta et al., 2023). The U.S. respondents showed a more positive attitude toward genome-edited food than the Japanese and German respondents. The risk perception by the Japanese respondents was similar to that by the German respondents, but the benefit perception by the Japanese respondents were closer to that by the U.S. respondents. These results suggested that regarding the acceptance of genome-edited food, Japanese people are between Americans and Germans. In 2021, after the MAFF accepted the notification of tomato #87-17, Shigi and Seo investigated Japanese attitudes toward genome-edited foods (Shigi and Seo, 2023). Their internet survey indicated that the respondents’ awareness influenced the information’s credibility, but not the perceived usefulness which was closely related to the information credibility. Moreover, less credibility of information and less perceived usefulness affected the respondents’ willingness to purchase genome-edited foods. Together, it was indicated that Japanese consumers demand more information about how genome-edited foods are produced and what their usefulness is.

**TABLE 2:** Survey results of Japanese attitudes to genome editing (GE) and genome-edited food (GEF).

Publication	Survey implementation	Method and subject	Major findings
Kato-Nitta et al. (2019)	From December 2016 to February 2017	Internet survey through an investigation company. Respondents:3,000 lay public, 111 molecular biology experts, and 86 experts in the other fileds	Three groups were compared in risk, benefit, and value perceptions of GE crops. The experts in other fields had benefit and value perceptions similar to molecular biology experts, while they had risk perception similar to lay public. Science literacy of lay public impacted their benefit perception but not risk or value perception of GE crops
Watanabe et al. (2020)	March 2016, January 2018, January 2019	Internet survey of a pre-registered sample of an investigation company. 3,100 respondents in 2016, 1,240 in 2018, and 1,543 in 2019	The scandal of genome-edited babies in China 2018 raised the people's awareness of GE; however, it also damaged GE reputation in Japan. Such medical uses of GE were not associated with agricultural uses of GE, although the awareness of both uses had risen
Tabei et al. (2020)	From May to October, 2019	Analysis of 14,066 Twitter* users and 28,722 tweets (posts): 2,536 original posts (8.8%), 326 replies to posts (1.1%), and 25,860 reposts (90%)	54.5% to 62.8% posts were negative about GEF and the Consumer Affairs Agency rules that do not require labelling but call for voluntary labelling. A strong demand for mandatory labeling of GEF was identified in posts; however, 90% of relevant posts were reposts
Shineha et al. (2024)	From February to April, 2020	Internet survey through an investigation company. Respondents: 4,000 public and 398 experts	Public: a “wait-and-watch” attitude toward GEF, high demand for basic information on GE, apprehension about proper risk governance systems for GEF, and trust in the scientific community were found. Experts: An emphasis on the adequacy and necessity of breeding by GE was observed
Kato-Nitta et al. (2021)	March 2020	Internet survey in 3 countries through investigation company. Respondents:1,842 in Japan, 1,962 in Germany, and 2,050 in the U.S.	U.S. respondents were the most positive attitudes toward GEF, and did not highly differentiate between GEF and conventionally bred food. Japanese respondents had attitudes similar to German respondents regarding risk perception, and closer to US respondents regarding benefit perception
Shigi and Seo (2023)	August 2021	Internet survey by authors. 550 respondents	Low awareness of GE was observed. Awareness of GE influenced information credibility but not usefulness perception in GEF. Usefulness perception was closely related to information credibility in GEF. Low information credibility and low usefulness perception significantly affected willingness to purchase GEF

*A social network service, Twitter was subsequently renamed ‘X'.

Those above-described surveys indicated that the majority of Japanese people are not sufficiently familiar with agricultural genome editing, although their awareness of genome editing has increased since the genome-edited baby scandal. Importantly, the same people express a desire to obtain information about genome editing and have some degree of concern about the safety of genome-edited foods. Those who are aware of genome editing are classified into two groups. One group credits information on the safety of and perceives the usefulness of genome-edited food crops, and the other group does not credit such information from experts, companies and the government and has deep concern about the potential risks of genome-edited food, calling for mandatory labeling, and gaining followers (particularly in the SNS).

## 5 Toward better consumer choices

Japan’s regulatory framework allowed domestic and international firms to receive official confirmations that their four genome-edited crop varieties are non-LMO and non-GM food (Table 1). Among the approved crop varieties, only Sanatech’s tomato #87-17 is sold online to consumers who credit safety information and perceive the usefulness of GABA. Tomato #87-17 seedlings were distributed for cultivation free of charge. In response to these, some consumers oppose genome-edited food crops. They call for mandatory labeling, while using their label of “OK seed” at stores to convey “not genome-edited”, which seems similar to campaigns against GM food. In contrast to those consumers, the majority of people are not sufficiently knowledgeable about genome-edited food crops, indicating that agricultural genome editing is not fully accepted in Japanese society. One of the reasons is that they have never even seen genome-edited crops at their stores. Social studies also underscore the need to hold sufficient communications about the potential risks of genome-edited crops because only science literacy does not impact people’s risk perception (Table 1). The use of genome editing may create sustainable and productive agriculture systems that benefit consumers, as well as producers (Anonymous). By sharing such visions in society, people’s awareness and credibility of safety information regarding agricultural genome editing can increase (Shigi and Seo, 2023). However, the Strategic Innovation Plan 2018 lacked such visions (Cabinet Office, 2018). Although the CAA held public meetings in five cities in July 2019, the meetings were designed to explain their rules regarding the partial deregulation of genome editing, not to discuss the visions of agricultural genome editing or to provide risk communications (CAA, 2019b). If the partial deregulation of genome editing had gone through legal amendments in the National Diet, such visions would have been deliberated and shared in Japanese society.

Nevertheless, it is not too late for Japan to hold risk communications regarding agricultural genome editing for mutual understanding among consumers, companies, experts and the government (Kato-Nitta et al., 2023). However, this premises that Japan’s risk governance system is credible among the general public (Shineha et al., 2024). It is important for Japan’s ministries to sufficiently clearly explain to people why food derived from SDN-1 and SDN-2 were deregulated from the Food Sanitation Law and that this is wider than the deregulation of SDN-1 from the Cartagena Law (Figure 1) to people. Moreover, Table 1 indicates that competent ministries assess, on a case-by-case basis, company’s data regarding the absence of exogenous DNA and off-target mutations in their products. Indeed, MOE stated that they may amend the deregulation of SDN-1 if any important knowledge regarding the safety of genome editing becomes available (Table 1). If exogenous DNA is overlooked in a genome-edited crop’s genome, the LMO crop is cultivated without being controlled by the Cartagena Law, potentially affecting biological diversity. Indeed, the overlooked insertion of introduced plasmid for genome editing has occurred abroad (Norris et al., 2020). Moreover, if an off-target mutation that produces hazardous substances, such as allergens, is overlooked in a genome-edited food product, consuming such a product might cause harm to one’s health. Because approximately 40% of Japanese have been concerned about potential risks of GM food (CAA, 2016) and because a majority of people want to know basic information about genome-edited food (Shineha et al., 2024), it is important to improve credibility in risk assessment of genome-edited food crops (Kato-Nitta et al., 2023; Shigi and Seo, 2023). Japan’s ministries should demand more rigorous assessment by combining multiple complementary assays with limitations to demonstrate the absence of exogenous DNA and off-target mutations in crop genomes (Ishii and Ishii, 2022), as done by Simplot (Table 1). Those ministries may also use artificial intelligence techniques, such as machine learning (Das et al., 2023), to enhance the review of data accuracy in genome-edited crops submitted by companies, although no such measures are currently planned in Japan.

When genome-edited food crops are sold at stores in the future, it is important for people to be aware of the genome-edited crops and then choose whether or not to purchase such food products. To achieve this awareness, genome-edited crops must be labeled as such, as illustrated in the genome-edited tomato #87-17. CAA should continue to call for voluntary labeling, emphasizing the importance of informing people of the usefulness and safety of genome-edited food (Shineha et al., 2024; Kato-Nitta et al., 2023; Shigi and Seo, 2023). Again, if any visions of agricultural genome editing are shared in society, CAA can persuasively call for genome-edited food labeling. Given that food labels convey information about the product’s identity and contents, the “not genome-edited” label, which some consumer groups use, appears to be undesirable. However, the MAFF currently withholds the exclusion of any genome-edited crops from organic agriculture (Figure 1). Consumers who oppose genome-edited food crops probably prefer organic agricultural products that rely on natural processes and avoid the use of synthetic inputs or methods. Because the MAFF’s unclear organic agriculture standards may contribute to the opposition to genome-edited crops, MAFF should promptly exclude genome-edited food crops from organic agriculture, as illustrated by for organic food standards in the Codex Alimentarius: “genetically engineered/modified organisms (GEO/GMO) are not compatible with the principles of organic production (either the growing, manufacturing, or processing) and therefore are not accepted” (WHO&FAO, 2007). In so doing, MAFF must discuss with MHLW and CAA about their deregulations of SDN-1 and 2 based on the indistinguishability between resultant genome-edited food and food produced by conventional breeding.

## 6 Conclusion

Since 2019, Japan has learned several lessons from introducing agricultural genome editing in society. To improve informed consumer choices regarding genome-edited food crops, it is essential to share the visions of agricultural genome editing throughout society, promote risk communication based on reliable risk governance, and maintain clear labels, including organic food standards. Such measures are critical for ensuring public trust and responsible adoption of genome-edited foods.
